# Comparison of open, laparoscopic, and robotic left colectomy for radical treatment of colon cancer: a retrospective analysis in a consecutive series of 211 patients

**DOI:** 10.1186/s12957-022-02796-8

**Published:** 2022-10-18

**Authors:** Zhixiang Huang, Taiyuan Li, Genghua Zhang, Zhen Zhou, Haoran Shi, Cheng Tang, Lingling Yang, Xiong Lei

**Affiliations:** 1 Gastrointernal Surgical Institute, Nanchang University, Nanchang, 330006 Jiangxi China; 2 China-Japan Union Hospital Of Jilin University, 130000 Chang Chun, China; 3 Department of General Surgery, The First Affiliated Hospital of Nanchang University, Nanchang, 330006 Jiangxi China; 4 Department of Gastroenterology, The Second Affiliated Hospital of Nanchang University, Nanchang, 330006 Jiangxi China

**Keywords:** Robotic surgery, Laparoscopic surgery, Open surgery, Left colectomy

## Abstract

**Background:**

Robotic surgery has been widely used in the radical treatment of colonic cancer. However, it is unclear what advantages the robotic approach offers over other approaches in left colectomy. This study aims to explore the advantage of robotic surgery in left colectomy by comparing open, laparoscopic, and robotic surgery.

**Methods:**

A retrospective analysis was performed on the clinical data of patients with radical left colectomy for colon cancer who were admitted to the Department of General Surgery, The First Affiliated Hospital of Nanchang University, from November 2012 to November 2017. Two hundred eleven patients included were divided into the open surgery group (OS, *n*=49), laparoscopic surgery group (LS, *n*=92), and robotic surgery group (RS, *n*=70) according to surgical techniques. The clinicopathologic data were collected for clinical outcome assessment. Finally, the clinical value of RS in radical left colectomy was further evaluated by propensity score matching (PSM) analysis.

**Results:**

Three groups were similar in demographics and clinical characteristics. Compared with OS, LS and RS groups had better intraoperative and perioperative clinical outcomes. Moreover, the RS group exhibited the minimum operative times, length of stay (LOS), and evaluated blood loss. LS and RS also exhibited less perioperative and postoperative long-term complications. Three groups showed similar postoperative pathological outcomes. The overall survival and disease-free survival were also similar among the three groups (all *P* > 0.05). Cox regression analysis showed surgical approach was not a prognostic factor for overall survival (*P* = 0.671) and disease-free survival (*P* = 0.776). PSM analysis of RS and LS by clinical characteristics showed RS showed shorter operation time (*P* < 0.001) and LOS for patients without complications (*P =* 0.005). However, no significant differences were found in perioperative and long-term postoperative complications, pathological outcomes, overall survival, and disease-free survival.

**Conclusions:**

Among three techniques for radical left colectomy, LS and RS had significant advantages over OS in short-term clinical outcomes, and no significant differences were found in overall, disease-free survival, local recurrence, and distant metastasis incidence. Moreover, RS shows better perioperative clinical outcomes but without compromising survival compared with LS.

**Supplementary Information:**

The online version contains supplementary material available at 10.1186/s12957-022-02796-8.

## Background

Colorectal cancer (CRC) is the second most deadly cancer and accounts for approximately 10% of all annually diagnosed cancers and cancer-related deaths worldwide [1]. In particular, left-sided colon cancer showed a worrying rise in young patients [2]. Surgical resection is still the only method to achieve radical treatment for resectable local left colonic cancer in the middle and advanced stages under elective conditions [3, 4]. Historically, OS has long been the most classic and useful surgical approach to treat left colonic cancer [5]. However, OS brings great trauma to the patients, such as long abdominal incision and severe postoperative pain, which is not conducive to patients’ postoperative recovery. Over the last several decades, minimally invasive surgery (MIS) including LS and RS has become more popular and has been adopted in colorectal surgery [6]. The studies showed MIS can decrease perioperative morbidity, facilitate accurate anatomical dissection, and improve the quality of the resection specimens [7, 8], but without compromising oncologic principles [9]. Results [10, 11] showed that LS has better cosmesis, shorter postoperative hospitalization, and faster recovery than OS. Recently, RS using the da Vinci surgical system shows several technical advantages [12] such as 3D visualization, elimination of fulcrum effect, multi-manipulator operation, and better ergonomic positioning and achieves similar or even better outcomes than LS in colorectal cancer [13, 14]. However, few investigations on surgery for radical left colectomy for colonic cancer are available according to current literature.

Our gastrointestinal center has evolved from open, laparoscopic, and robotic surgery for radical resection of CRC. In rectal cancer, our study revealed short-term advantages of RS, but without a beneficial effect on survival [15]. In this study, a comprehensive comparison of perioperative outcomes and survival in consecutive series of 211 patients who were treated by three different surgical techniques (open, laparoscopic, and robotic) for radical left colectomy, and the safety and efficacy were also analyzed. Moreover, the value of MIS, special for RS, in radical left colectomy was verified.

## Methods

### Study population and data collection

This is a retrospective study of patients undergoing radical left colectomy for colonic cancer under elective conditions from November 2012 to November 2017 at the largest gastrointestinal center in Jiangxi Province, The First Affiliated Hospital of Nanchang University. Detailed eligibility criteria are shown as follows:

The inclusion criteria are (1) tumor in the left side including the distal 1/3 of the transverse colon (TC), splenic flexure (SF), the upper segment of descending colon (UDC), or the lower segment of descending colon (LDC); (2) age older than 18 and younger than 80 years; (3) primary colonic adenocarcinoma confirmed pathologically by endoscopic biopsy; (4) pathologic T1-4aN0-2M0 at postoperative evaluation according to 8th AJCC Cancer Staging Manual; (5) no history of malignancy in other organs; (6) ASA scores I, II, or III; and (7) written informed consent.

The exclusion criteria are (1) age>80 and <18 years; (2) with other malignant tumors; (3) TNM stage at 0, IV; (4) multi-visceral resection (tumors invading adjacent organs or tumors located in multiple bowel tubes); (5) palliative surgery; (6) history of CRC surgery; and (7) emergency surgery due to complications (bleeding, obstruction, or perforation) caused by left-sided colon cancer.

Eligible patients received routine preoperative chest X-ray or CT scan, abdominal CT scan, tumor markers, colonoscopy, and other liver ultrasound or MRI. All patients were assigned to robotic, laparoscopic, and open surgical procedure groups according to local medical insurance and the intention-to-treat principle. Some cases with LS and RS were converted to the OS due to intraperitoneal adhesions and were included in the OS group if met with inclusion criteria. Moreover, to reduce potential bias caused by the limitations of a retrospective cohort study, the propensity score matching (PSM) method was used to conduct a 1-to-1 matching analysis between the LS group and the RS group.

### Surgical treatment and follow-up

All surgical operations were performed by surgeons of similar experience and seniority. Surgical indication followed the guide treatment of colorectal cancer [16]. The length of bowel resection and scope of the lymph node dissection for radical left colectomy that was treated, the distal 1/3 of the transverse colon (TC), splenic flexure (SF), the upper segment of descending colon (UDC), or the lower segment of descending colon (LDC) are shown in the Supplementary Fig 1. The surgery was to remove the tumor-bearing colon segment and its corresponding mesocolon and ligate the origin of the inferior mesenteric artery (IMA) to maximize lymph node dissection (LND) without damaging the visceral fascia layer. Our surgical team attempted to secure 10 cm or more for the proximal and distal resection margin and followed D3 lymphadenectomy [17] and complete mesocolic excision (CME) principles [18]. The procedures for radical left colectomy were as follows: firstly, dissociated inferior mesenteric artery (IMA) and then ligated the origin of the left colon artery (LCA) while preserving the superior rectal artery and part of the sigmoid artery. Secondly, the mesocolon was incised along the inferior pancreatic border, transecting the left colic vein or inferior mesenteric vein (IMV), and the left branch of the middle colic artery. Thirdly, the division of the splenocolic and gastrocolic ligament and the lateral peritoneal fold completed the mobilization of the splenic flexure and accomplished the CME; Forthly, the mobilized left colon was removed to accomplish side-to-end or side-to-side stapled extracorporeal or intracorporeal anastomosis; Lastly, final operations were to flush the abdominal cavity, place a drainage tube after inspection, and suture the auxiliary incision.

All patients were regularly followed-up after surgery. A follow-up visit involved standard clinical examination, serum tumor marker test, and imaging by computer tomography (CT) or/and magnetic resonance or/and ultrasounds. Patients were treated with adjuvant chemotherapy (CHT) when indicated.

### Definitions

Comorbidity was measured using the Charlson Comorbidity Index (CCI) and selected elements of Elixhauser comorbidity score (cardiovascular, pulmonary, endocrine, gastrointestinal, renal, inflammatory, and neuropsychiatric disorders) [19].

The conversion was defined as the use of a midline or periumbilical long incision for any reason at any time during the procedure. Cases, converted to OS, who met the inclusion criteria, were to the OS group.

Postoperative complications were stratified by the Clavien–Dindo classification of surgical complications [20]. Adverse events were defined as any deviation from the normal postoperative course, and major adverse events were defined as any event that is life-threatening, requires inpatient hospitalization, results in a single organ or multiorgan failure, or requires operative, endoscopic, or radiological intervention [21]. Major adverse events correspond to Grade III/IV/V of the Clavien–Dindo classification [20, 22]. Long-term complications were defined as postoperative complications that occurred after the 30th postoperative day. The short-term clinical outcomes included intraoperative and perioperative outcomes, perioperative postoperative complications, and postoperative pathologic outcomes. Then, long-term postoperative complications and survival outcomes were incorporated into long-term clinical outcomes.

Overall survival was calculated in months from the time of surgery to the last follow-up or death. Recurrence was defined as the presence of locoregional recurrence, the presence of distant metastases, or death from colorectal cancer. Locoregional recurrence was defined as the relapse of the tumor at the primary site confirmed by radiological or histological evidence. Distant metastasis was considered as metastatic lesions that were diagnosed in other organs beyond the primary site.

### Statistical analysis

Categorical variables were presented as counts and percentages. Normally distributed continuous variables were expressed as mean ± standard deviations (SD). Comparisons of categorical variables were performed using Fisher’s exact test or *χ*^2^ test, whereas comparisons of quantitative variables were carried out using Kruskal-Wallis tests or Wilcoxon rank sum tests. A matched comparative analysis between the LS and RS groups was conducted based on propensity score matching (PSM, a logistic regression model with a match tolerance value of 0.01) by one-to-one nearest-neighbor matching with covariates as follows: age, sex, BMI, ASA score, previous abdominal surgery, smoking and drinking history, family history of CRC, CEA, lymph node metastasis, CCI score, with comorbidities, postoperative chemotherapy, tumor location, tumor differentiation, and pTNM stage. The overall survival (OS), disease-free survival (DFS) rates, and cumulative incidence of local recurrence (LR) and distant metastasis were calculated by using the Kaplan–Meier method and compared by log-rank test. All statistically significant factors determined by univariate analysis were then analyzed by multivariate analysis using the Cox proportional hazards regression model. PSM and all statistical analyses were conducted using SPSS 26.0 (SPSS Inc., Chicago IL, USA). *P*<0.05 was set as the criterion for statistical significance.

## Results

### Demographics and clinical characteristics

**Fig. 1:**
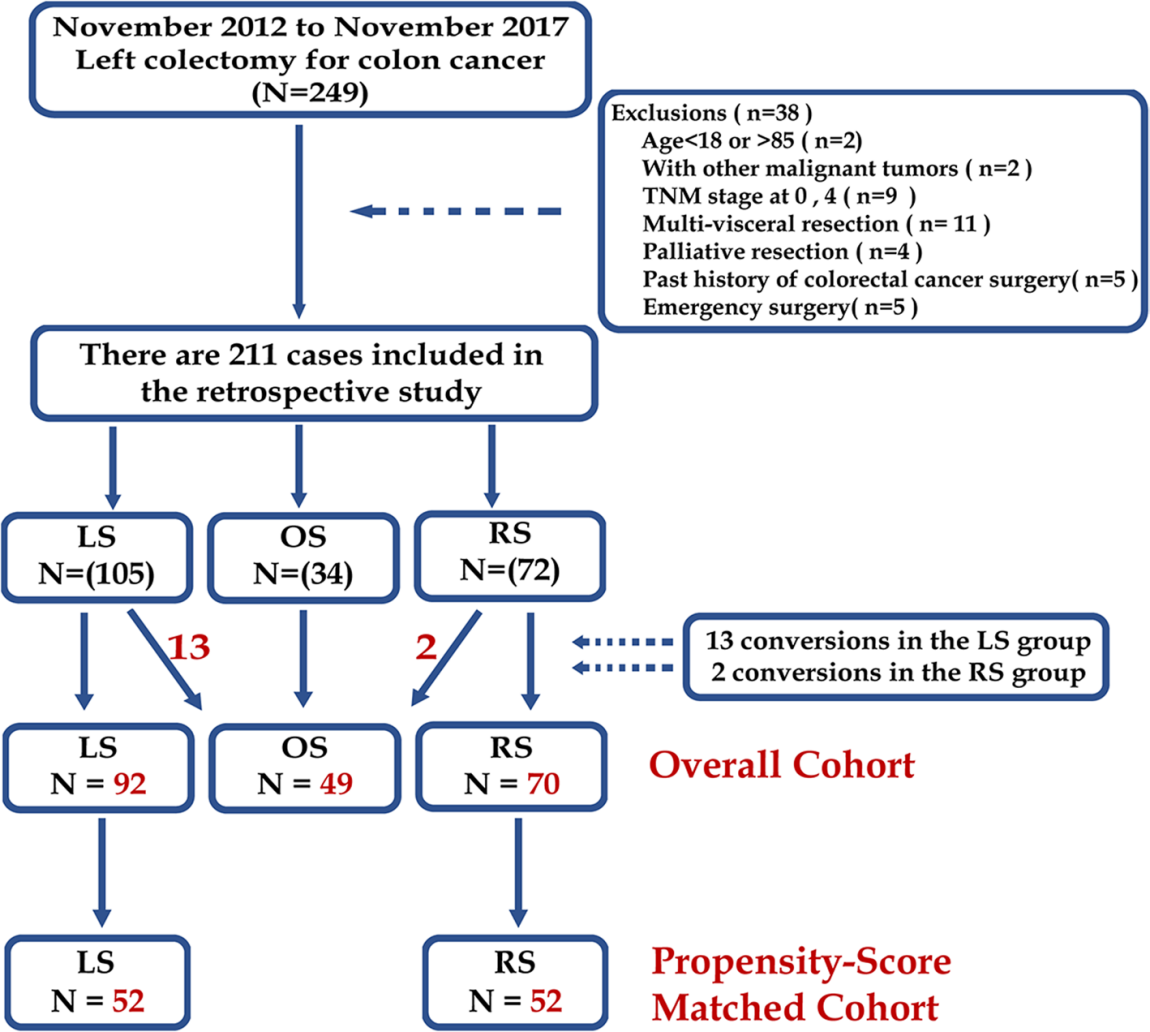
Flow diagram of case selection

A total of 211 patients met the inclusion criteria and were evaluated in three groups (Fig. 1). Due to tumor with advanced T stage, severe peripheral adhesion and anatomical ambiguity, 13 of 105 patients originally scheduled for LS and 2 of 72 in the RS group were converted to OS. A statistical difference was found in conversion rates between the LS and RS groups (12.4 vs. 2.8%; *P* = 0.028). The demographics and clinical characteristics of the three groups were shown no significant differences among groups in age, sex, BMI, ASA score, smoking and drinking history, family history of CRC, lymph node metastasis, and preoperative comorbidities among three groups (all *P >* 0.05; Supplementary Table 1). Though no significance was revealed, cases with previous abdominal surgery in OS (32.7%) were more than that in LS (18.5%) and RS (17.1%). The average preoperative number of red blood cells in OS was significantly highest (*P* = 0.022; Supplementary table 1), but no significance was shown between RS and LS groups (*P* = 0.798; Supplementary table 1). Preoperative SEMS insertion and postoperative adjuvant chemotherapy were similar among the three groups (*P >* 0.05; Supplementary table 1).

### Intraoperative and perioperative clinical outcomes

Operative time in the RS group was 141.4±40.0 min and was the shortest among the three groups (*P <* 0.001; Table 1), but no difference between the LS group (186.1±43.7 min) and the OS group (175.9±43.6 min). MIS including LS and RS exhibited less blood loss and shorter length of hospital stay than OS (*P <* 0.05; Table 1), while RS had the least blood loss (127.6±70.0ml) and shortest length of hospital stay (7.7±3.1 days). The time to first bowel movement, first flatus, and first liquid diet was the significantly longest in the OS group (*P <* 0.001; Table 1), but no difference was observed between LS and RS (*P* > 0.05; Table 1). Except for WBC, hematology examination, including RBC, TP, and ALB, showed significant differences between pre- and post-operation in three groups (all *P* < 0.001; Supplementary Fig. 2A1&B1&C1&D1). However, the changes from pre-operation to post-operation in RBC (*P* = 0.373, Supplementary Fig. 2A2), WBC (*P* = 0.747, Supplementary Fig. 2B2), TP (*P* = 0.210, Supplementary Fig. 2C2), and ALB (*P* = 0.112, Supplementary Fig. 2D2) were similar among three groups (Supplementary Fig. 2).

**Table 1:** Intraoperative and perioperative clinical outcomes (overall cohort)

Variables	OS *N*=49	LS *N*=92	RS *N*=70	*P*
Operation time (min)	175.9±43.6	186.1±43.7	141.4±40.0	**<0.001**^**§**^
			RS vs OS; ***P*<0.001** RS vs LS; ***P*<0.001** LS vs OS; *P=*0.646	
Blood loss (ml)	223.6±143.2	156.4±79.8	127.6±70.0	**<0.001**^**§**^
			RS vs OS; ***P*<0.001** RS vs LS; ***P=*0.028** LS vs OS; ***P=*0.012**	
Time to first bowel movement (h)	60.0±12.2	39.6±16.8	33.6±16.3	**<0.001**^**§**^
			RS vs OS; ***P*<0.001** RS vs LS; *P=*0.126 LS vs OS; ***P*<0.001**	
Time to first flatus (h)	78.5±19.4	65.5±16.8	63.6±9.6	<0.001^**§**^
			RS vs OS; ***P*<0.001** RS vs LS; *P=*0.674 LS vs OS; ***P=*0.001**	
Time to first liquid diet (h)	105.5±15.8	88.4±21.3	81.6±14.4	**<0.001**^**§**^
			RS vs OS; ***P*<0.001** RS vs LS; *P=*0.119 LS vs OS; ***P*<0.001**	
LOS for all patients (d)	9.8±3.8	8.7±4.8	7.7±3.1	**<0.001**^**§**^
			RS vs OS; ***P*<0.001** RS vs LS; ***P=*0.002** LS vs OS; ***P=*0.002**	
LOS for patients without complications (d)	8.1±1.3	7.2±0.7	6.7±0.8	**<0.001**^**§**^
			RS vs OS; ***P*<0.001** RS vs LS; ***P=*0.018** LS vs OS; ***P=*0.004**	
LOS for patients with complications (d)	13.2±4.8	14.5±8.3	14.5±5.4	0.754^**§**^

Values were expressed as mean (SD = standard deviation) or *n* (%)

*Abbreviation*: *RS* Robotic surgery, *LS* Laparoscopic surgery, *OS* Open surgery, *LOS* Length of stay

^**§**^Kruskal-Wallis test

### Perioperative and long-term postoperative complications

No deaths, reoperations, and readmissions happened in the LS and RS groups (all *P* > 0.05; Table 2), but one death was for septic shock in the OS group. Overall perioperative morbidity was the highest in the OS group (38.8%; Table 2), but no significant difference was found between RS and LS (19.6 vs*.* 11.4%; *P* > 0.05; Table 2). According to Clavien–Dindo grade, the OS group still had the highest incidence in grade III/IV complications (*P* = 0.004; Table 2). No significant differences were observed in the occurrence of overall and major long-term complications (all *P* > 0.05; Table 2). However, the rate of incisional hernia in OS was the highest (*P* = 0.036; Table 2).

**Table 2:** Perioperative and long-term postoperative complications (overall cohort)

Variables	Open *N*=49	Laparoscopic *N*=92	Robotic *N*=70	*P*
**Perioperative complications**^**a**^				
Mortality, *n* (%)	1 (2.0%)	0 (0.0%)	0 (0.0%)	0.232^**‡**^
Reoperation, *n* (%)	0 (0.0%)	0 (0.0%)	0 (0.0%)	/
Readmission, *n* (%)	0 (0.0%)	0 (0.0%)	0 (0.0%)	/
Overall morbidity, *n* (%)	19 (38.8%) z	18 (19.6%) y	8 (11.4%) y	**0.001**^**†**^
Grade I/II complications, *n* (%)	13(26.5%)	17 (18.5%)	8 (11.4%)	0.107^**†**^
Wound infection	4 (8.2%)	6 (6.5%)	5(7.1%)	0.942^**‡**^
Intra-abdominal infection	0 (0.0%)	2 (2.2%)	0 (0.0%)	0.506^**‡**^
Ileus	0 (0.0%)	2 (2.2%)	1 (1.4%)	0.796^**‡**^
Acute pneumonia	1 (2.0%)	1 (1.1%)	1 (1.4%)	1.000^**‡**^
Fever of unknown origin	4 (8.2%) z	3 (3.3%) zy	0 (0.0%) y	**0.037**^**‡**^
Anastomotic hemorrhage	0 (0.0%)	1 (1.1%)	0 (0.0%)	1.000^**‡**^
Anastomotic leak	0 (0.0%)	1 (1.1%)	0 (0.0%)	1.000^**‡**^
Blood transfusion due to anemia	4 (8.2%) z	1 (1.1%) y	1 (1.4%) y	**0.049**^**‡**^
Grade III/IV complications, *n* (%)	5 (10.2%) z	1 (1.1%) y	0 (0.0%) y	**0.004**^**‡**^
Wound dehiscence (fascia)	1 (2.0%)	0 (0.0%)	0 (0.0%)	0.232^**‡**^
Intra-abdominal infection and effusion	4 (8.2%) z	0 (0.0%) y	0 (0.0%) y	**0.003**^**‡**^
Ileus	0 (0.0%)	0 (0.0%)	0 (0.0%)	1.000^**‡**^
Acute liver failure	0 (0.0%)	1 (1.1%)	0 (0.0%)	1.000^**‡**^
**Long-term postoperative complications**^**b**^				
Overall morbidity, *n* (%)	5 (10.2%)	3 (3.3%)	2 (2.9%)	0.133^**‡**^
Grade I/II complications, *n* (%)	1 (2.0%)	1 (1.1%)	1 (1.4%)	1.000^**‡**^
Incisional hernia	0 (0.0%)	1 (1.1%)	0 (0.0%)	1.000^**‡**^
Ileus	1 (2.0%)	0 (0.0%)	1 (1.4%)	0.317^**‡**^
Grade III/IV complications, *n* (%)	4 (8.2%)	2 (2.2%)	1 (1.4%)	0.160^**‡**^
Incisional hernia	2 (4.1%) z	0 (0.0%) y	0 (0.0%) y	**0.036**^**‡**^
Adhesion	2 (4.1%)	1 (1.1%)	0 (0.0%)	0.171^**‡**^
Anastomotic stricture	0 (0.0%)	1 (1.1%)	1 (1.4%)	1.000^**‡**^
Major perioperative complications, *n* (%)	6 (12.2%) z	1 (1.1%) y	0 (0.0%) y	**0.001**^**‡**^
Major long-term complications, *n* (%)	4 (8.2%)	2 (2.2%)	1 (1.4%)	0.160^**‡**^

Values are expressed as *n* (%)

Major complications were defined as complications with a Grade III and higher of the Clavien–Dindo classification. Different letters (z and y) marked on the right side of the cell frequency indicated that there is a statistically different between the two groups that is the probability of the significance test for the comparison between the two groups is less than 0.05 (*P* < 0.05)

^**†**^Pearson’s chi-squared test

^**‡**^Fisher’s exact test

^a^Complications within 30 days from operation date

^b^New complications 30 days after operation date

### Postoperative pathological outcomes

There were no significant differences among the three groups in tumor location, with adenomatous polyps, neoplasm diameter, tumor differentiation, pTNM stage, lymph node metastasis, lympho-vascular invasion, and perineural invasion (all *P* > 0.05; Table 3). In addition, the number of retrieved lymph nodes and distal resection margin showed no statistical difference (all *P* > 0.05; Table 3). No positive margin was detected in the three groups (Table 3).

**Table 3:** Postoperative pathologic outcomes (overall cohort)

Variables	Open *N*=49	Laparoscopic *N*=92	Robotic *N*=70	*P*
Tumor location				0.067^**†**^
TC&SF, *n* (%)	14 (28.6%)	26 (28.3%)	16 (22.9%)	
UDC, *n* (%)	24 (49.0%)	29 (31.5%)	21 (30.0%)	
LDC, *n* (%)	11 (22.4%)	37 (40.2%)	33 (47.1%)	
With adenomatous polyps, *n* (%)	11 (22.4%)	31 (33.7%)	14 (20.0%)	0.112^**†**^
Neoplasm longest diameter (cm)	5.6±2.4	5.1±1.8	5.0±1.8	0.243^**§**^
Tumor differentiation				0.834^**‡**^
Well, *n* (%)	3 (6.1%)	10 (10.9%)	8 (11.4%)	
Moderate, *n* (%)	36 (73.5%)	69 (75.0%)	54 (77.1%)	
Poor, *n* (%)	7 (14.3%)	8 (8.7%)	5 (7.1%)	
Mucinous, *n* (%)	3 (6.1%)	5 (5.4%)	3 (4.3%)	
pTNM stage				0.261^**†**^
I, *n* (%)	5 (10.2%)	14 (15.2%)	15 (21.4%)	
II, *n* (%)	22 (44.9%)	45 (48.9%)	24 (34.3%)	
III, *n* (%)	22 (44.9%)	33 (35.9%)	31 (44.3%)	
pT stage				0. 412^**‡**^
T1, *n* (%)	2 (4.1%)	10 (10.9%)	10 (14.3%)	
T2, *n* (%)	4 (8.2%)	4 (4.3%)	5 (7.1%)	
T3, *n* (%)	6 (12.2%)	11 (12.0%)	4 (5.7%)	
T4a, *n* (%)	35 (75.5%)	62 (72.8%)	51 (72.9%)	
pN stage				0.399^**†**^
N0, *n* (%)	27 (55.1%)	59 (64.1%)	39 (55.7%)	
N1, *n* (%)	13 (26.5%)	21 (22.8%)	24 (34.3%)	
N2, *n* (%)	9 (18.4%)	12 (13.0%)	7 (10.0%)	
Number of lymph node harvest	14.7±6.2	14.0±6.0	14.0±4.8	0.725^**§**^
Positive resection margin, *n* (%)	0 (0.0%)	0 (0.0%)	0 (0.0%)	/
With lymph node metastasis, *n* (%)	22 (44.9%)	32 (34.8%)	31 (44.3%)	0.358^**†**^
With lymphovascular invasion, *n* (%)	11 (22.4%)	14 (15.2%)	10 (14.3%)	0.447^**†**^
With extranodal tumor deposits, *n* (%)	6 (12.2%)	14 (15.2%)	13 (18.6%)	0.639^**†**^
With perineural invasion, *n* (%)	17 (34.7%)	34 (37.0%)	24 (34.3%)	0.931^**†**^

Values are expressed as mean (SD = standard deviation) or *n* (%)

*Abbreviation*: *TC* The distal 1/3 of the transverse colon, *SF* Splenic flexure, *UDC* Upper segment of descending colon, *LDC* Lower segment of descending colon, *pTNM* Pathological tumor-node-metastasis

^**§**^Kruskal-Wallis test

^**†**^Pearson’s chi-squared test

^**‡**^Fisher’s exact test

### Survival analyses and prognostic factors

**Fig. 2:**
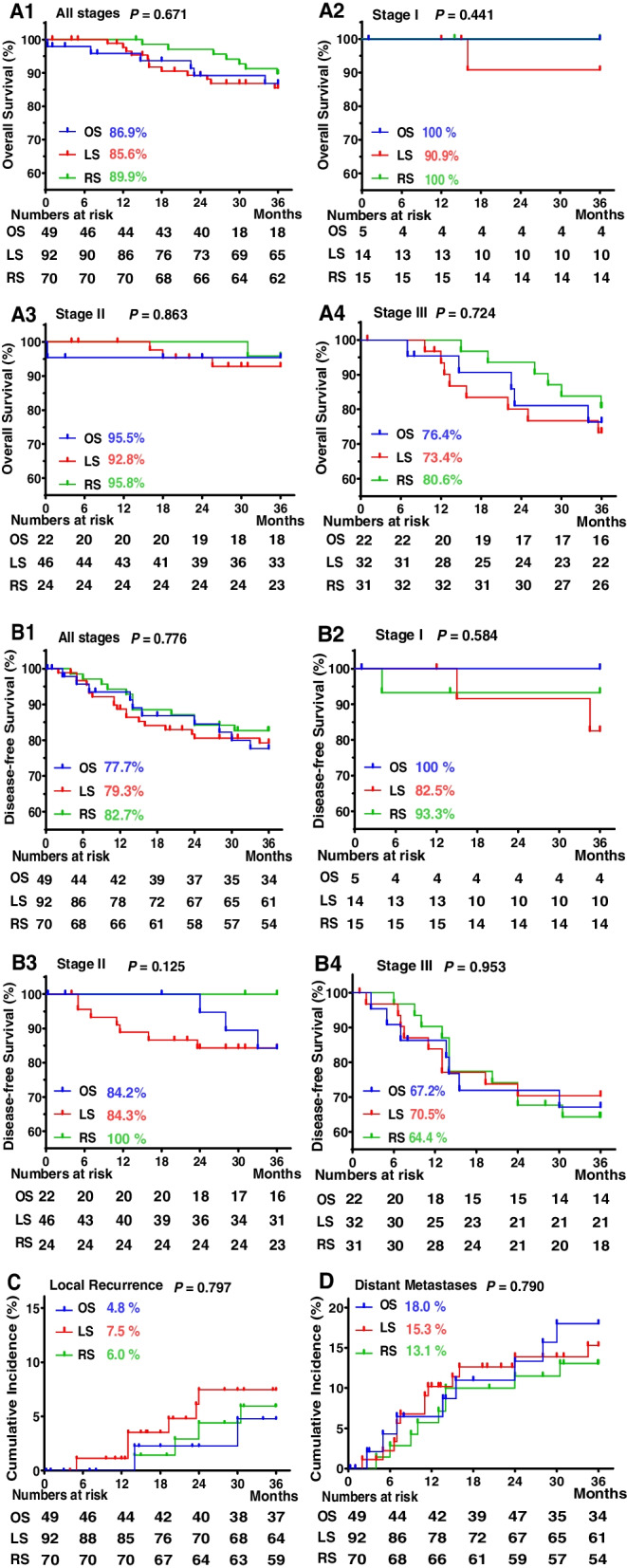
Kaplan–Meier survival curves and cumulative incidence curves (overall cohort). **A** Kaplan–Meier survival curves for overall survival rates according to TNM stage. A1 All stages, A2 Stage I, A3 Stage II, and A4 Stage III. **B** Kaplan–Meier survival curves for disease-free survival rates according to TNM stage. B1 All stages, B2 Stage I, B3 Stage II, and B4 Stage III. **C** Cumulative incidence curves of local recurrence rates. **D** Cumulative incidence curves of distant metastasis rates

The median follow-up period was 40 months for the overall population. No statistical differences were observed in overall survival among the three groups (*P* = 0.671; Fig. 2A1). Also, no statistical difference was found in the overall survival by subtype analysis according to the TNM stage among three groups (all *P* > 0.05; Fig. 2A2, A3, and A4). Similarly, the disease-free survival rate showed no significant differences among the three groups (*P* = 0.671; Fig. 2B1). Subtype analysis of the disease-free survival still showed no significant difference according to the TNM stage among the three groups (all *P* > 0.05; Fig. 2B1, B2, and B3). Cumulative local recurrence rate (*P* = 0.797; Fig. 2C) and cumulative distant metastatic incidence (*P* = 0.790; Fig. 2D) was similar among three groups (Fig. 2).

In univariate analysis, factors that affected both overall and disease-free survival were postoperative adjuvant chemotherapy, tumor differentiation, pTNM stage, pN stage, number of lymph node harvest, lymphovascular invasion, extranodal tumor deposits, and perineural invasion (all *P* < 0.05; Table 4). The local recurrence rate was affected by three risk factors such as the level of serum CEA> 6.5, pN stage, and perineural invasion (all *P* < 0.05; Table 4). However, surgical approaches were not prognostic factors for overall and disease-free survival and local recurrence rates in this study (all *P* < 0.05; Table 4). In multivariate analysis, the prognostic factors for the overall survival were postoperative adjuvant chemotherapy (HR [CI] 2.407 (1.056–5.483); *P* = 0.037; Table 5), pN stage (HR [CI] 3.251 (1.856–5.695); *P* < 0.001; Table 5), and prognostic factors for disease-free survival were CEA (HR [CI] 2.036 (1.069–3.877); *P*=0.030; Table 5), and pN stage (HR [CI] 2.516 (1.689–3.747); *P* < 0.001; Table 5). Perineural invasion was the only prognostic risk factors for local recurrence (HR [CI] 9.275 (2.014–42.721); *P*=0.004; Table 5).

**Table 4:** Prognostic factors of a 3-year survival and local recurrence by univariate analysis (overall cohort)

Variables	*N*=211	Overall survival (%)	P*	Disease-free survival (%)	P*	Cumulative local recurrence (%)	P*
Age (years)			0.707		0.809		0.410
≤65	132	88.1		79.9		7.4	
> 65	79	86.0		80.2		4.4	
Sex			0.090		0.898		0.312
Male	124	90.6		79.9		7.8	
Female	87	82.5		80.4		3.9	
BMI (kg/m^2^)			0.364		0.255		0.822
≤25	169	86.2		78.4		6.6	
>25	42	92.1		86.9		5.4	
ASA score			0.385		0.144		0.373
1	1	100.0		100.0		0.0	
2	85	83.9		75.8		9.7	
3	125	89.5		85.5		4.3	
CEA (ng/ml)			0.056		**0.001**		**0.010**
≤6.5	164	89.7		84.7		4.0	
> 6.5	47	78.6		63.5		15.5	
CA199 (ng/ml)			**<0.001**		**0.015**		0.124
≤27	182	90.7		82.4		5.4	
> 27	29	64.5		63.0		13.5	
Surgical approach			0.671		0.776		0.797
Open	49	86.9		77.7		4.8	
Laparoscopy	92	85.6		79.3		7.5	
Robot	70	89.9		82.7		6.0	
Previous abdominal surgery			0.528		0.307		0.285
Yes	45	90.2		85.5		9.6	
No	166	86.6		78.6		5.4	
Smoking and drinking history			0.433		0.222		0.155
Yes	66	90.1		74.5		4.6	
No	145	86.1		82.6		10.1	
Family history of CRC			0.285		0.637		0.233
Yes	21	95.0		85.2		0.0	
No	190	86.4		79.5		7.1	
With comorbidities			0.264		0.906		0.775
Yes	80	83.4		80.0		5.6	
No	131	89.7		80.1		6.8	
Postoperative adjuvant chemotherapy			**<0.001**		**<0.001**		0.220
Yes	47	70.6		85.0		10.2	
No	164	92.1		62.9		5.4	
Perioperative morbidity			0.963		0.758		0.740
Yes	45	87.8		77.8		5.4	
No	166	87.2		80.6		6.6	
Tumor location			0.821		0.542		0.511
TC&SF	50	89.2		85.6		4.4	
UDC	74	85.6		76.8		4.8	
LDC	87	87.7		79.6		8.7	
With adenomatous polyps			**0.009**		0.067		0.137
Yes	56	98.0		88.6		1.8	
No	155	83.6		77.0		8.1	
Neoplasm diameter (cm)			0.395		0.816		0.089
≤5	136	85.8		80.7		4.2	
>5	75	89.9		79.0		10.2	
Tumor differentiation			**0.022**		**0.002**		0.089
Well	21	100.0		89.7		0.0	
Moderate	159	88.5		83.4		5.5	
Poor	20	74.4		59.2		13.8	
Mucinous	11	70.0		50.5		22.2	
pTNM stage			**<0.001**		**<0.001**		**0.030**
I	34	96.6		90.1		0.0	
II	91	95.3		89.5		3.6	
III	86	75.9		66.4		11.9	
pT stage			0.067		0.148		0.434
T1	22	100.0		95.0		0.0	
T2	13	100.0		91.7		0.0	
T3	21	95.2		85.4		5.0	
T4a	155	83.5		76.4		7.9	
pN stage			**<0.001**		**<0.001**		**0.007**
N0	125	95.7		89.6		2.7	
N1	58	87.5		74.9		9.1	
N2	28	52.0		48.9		19.4	
Number of lymph nodes detected			0.395		0.856		0.306
≤15	137	85.8		79.8		5.0	
>15	74	89.9		80.4		8.8	
With lymphovascular invasion			**<0.001**		**0.001**		0.059
Yes	35	69.9		59.8		14.8	
No	176	90.8		83.9		4.9	
With extranodal tumor deposits			**0.004**		**<0.001**		0.331
Yes	33	72.5		57.3		10.9	
No	178	90.3		84.5		5.6	
With perineural invasion			**0.001**		**<0.001**		**<0.001**
Yes	75	77.1		65.9		15.9	
No	136	92.8		87.7		1.6	

*Abbreviation*: *BMI* Body mass index, *ASA* American Society of Anesthesiology, *CRC* Colorectal cancer, *TC* The distal 1/3 of the transverse colon, *SF* Splenic flexure, *UDC* Upper segment of descending colon, *LDC* Lower segment of the descending colon, *pTNM* Pathological tumor-node-metastasis

*The 3-year overall and disease-free survival rates and cumulative local recurrence were calculated by using the Kaplan–Meier method

**Table 5:** Prognostic factors of a 3-year survival and local recurrence by multivariate analysis (overall cohort)

Variables	Overall survival HR (95% CI)	P*	Disease-free survival HR (95% CI)	P*	Cumulative local recurrence HR (95% CI)	P*
CEA	/	/	2.036 (1.069-3.877)	**0.030**	/	0.059
CA199	/	0.062	/	0.946	/	—
Postoperative adjuvant chemotherapy	2.407 (1.056-5.483)	**0.037**	/	0.067	/	—
With adenomatous polyps	/	0.058	/	/	/	—
Tumour differentiation	/	0.125	/	0.103	/	—
pN stage	3.251 (1.856-5.695)	**<0.001**	2.516 (1.689-3.747)	**<0.001**	/	0.233
With lymphovascular invasion	/	0.369	/	0.122	/	—
With extranodal tumor deposits	/	0.684	/	0.383	/	—
With perineural invasion	/	0.277	/	0.079	9.275 (2.014–42.721)	**0.004**

CI indicates confidence interval; *HR*, hazard ratio

*Cox proportional hazards regression model

### Propensity score matching analysis between LS and RS

As the above results showed minimally invasive surgery (MIS) approaches including LS and RS showed better perioperative outcomes but without compromising oncologic principles, the advantage of LS or RS in left colectomy still needs to be validated. To this end, 52 cases in the RS group and 52 cases in the LS group were included after one-to-one nearest-neighbor propensity score matching (PSM) analysis with multiple covariates (all *P* > 0.05; Supplementary Table 2). Compared with LS for left colectomy after PSM, RS showed a shorter operation time (LS 188.3±46.0 min vs. RS 149.2±41.7 min, *P* < 0.001; Supplementary Table 3) and LOS for patients without complications (LS 7.2±0.7 days vs. RS 6.8±0.8 days, *P* = 0.005; Supplementary Table 3), but both exhibited similar perioperative and long-term postoperative complications (all *P* > 0.05; Supplementary Table 4).

**Fig. 3:**
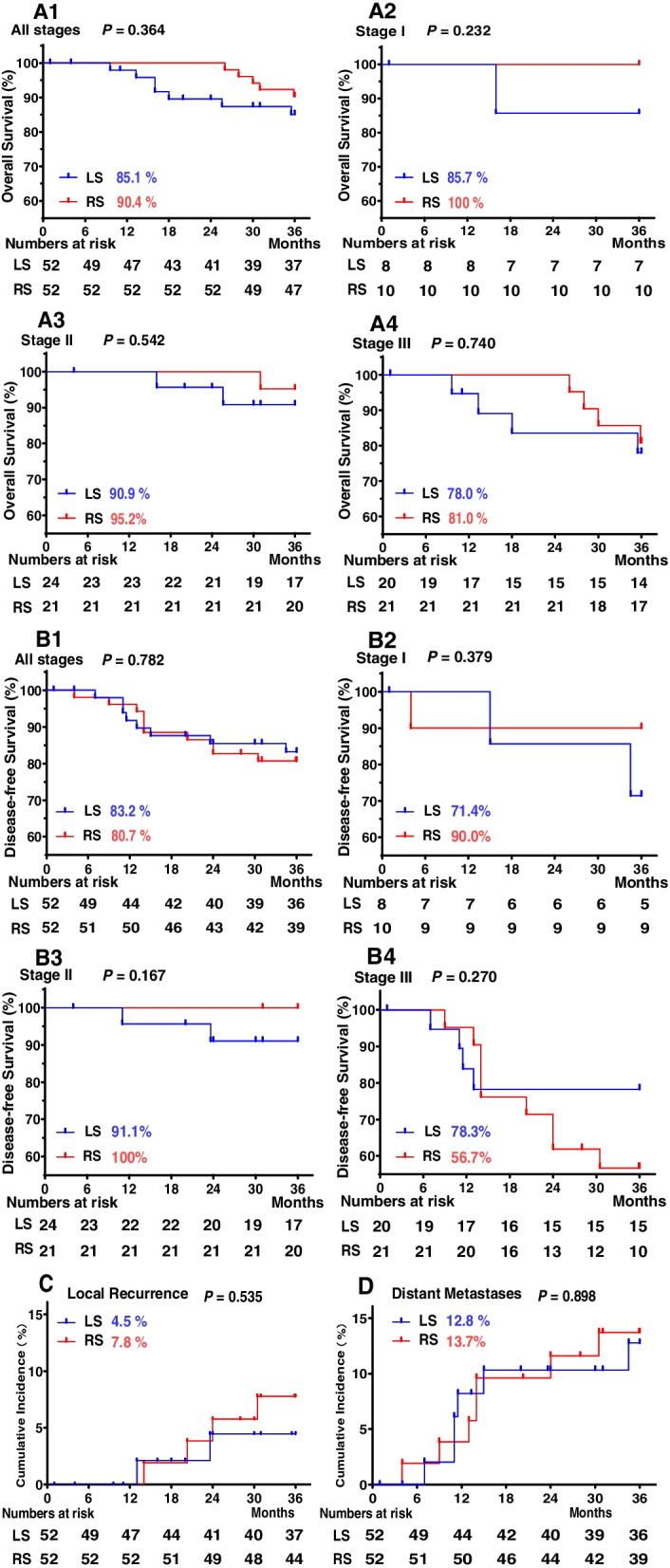
Kaplan–Meier survival curves and cumulative incidence curves (propensity score-matched cohort). **A** Kaplan–Meier survival curves for overall survival rates according to TNM stage. A1 All stages, A2 Stage I, A3 Stage II, and A4 Stage III. **B** Kaplan–Meier survival curves for disease-free survival rates according to the TNM stage. B1 All stages, B2 Stage I, B3 Stage II, and B4 Stage III. **C** Cumulative incidence curves of local recurrence rates. **D** Cumulative incidence curves of distant metastasis rates

Before PSM, the result of survival analysis showed no statistical difference was found in the overall survival (*P* = 0.367; Supplementary Fig. 3A1) and by subtype analysis according to TNM stage between LS and RS (all *P* > 0.05; Supplementary Fig. 3A2, A3, and A4). Similarly, the disease-free survival rate showed no significant differences between LS and RS (*P* = 0.544; Supplementary Fig. 3B1). Subtype analysis of the disease-free survival still showed no significant difference at stage I (*P* = 0.472; Supplementary Fig. 3B2) and III (*P* = 0.767; Supplementary Fig. 3B4) according to the TNM stage between LS and RS. However, the disease-free survival of RS was longer than that of LS at stage II (*P* = 0.044; Supplementary Fig. 3B3) between LS and RS. Cumulative local recurrence rate (*P* = 0.665; Supplementary Fig. 3C) and cumulative distant metastatic incidence (*P* = 0.679; Supplementary Fig. 3D) were similar between LS and RS (Supplementary Fig. 3). After PSM, the result of survival analysis showed overall survival (all *P* > 0.05; Fig. 3A), and disease-free survival (all *P* > 0.05; Fig. 3B) was paralleled between RS and LS groups according to the TNM stage. The similar results were for cumulative incidence of local recurrence (*P* = 0.535, Fig. 3C) and distant metastasis (*P* = 0.898, Fig. 3D) between LS and RS.(Fig. 3) Univariate and then multivariate Cox regression analysis showed the prognostic factors for overall survival were tumor differentiation (HR [CI] 2.007 (1.009–3.993); *P* =0.047) and pN stage (HR [CI] 2.920 (1.136–7.509); *P* = 0.026). The prognostic factors for disease-free survival were tumor differentiation (HR [CI] 2.665 (1.392–5.101); *P* =0.003), pN stage (HR [CI] 3.211 (1.154–8.934); *P* = 0.025) and extranodal tumor deposits (HR [CI] 3.881 (1.046–14.403); *P* = 0.043). The prognostic factors for cumulative local recurrence were tumor differentiation (HR [CI] 3.248 (1.203–8.772); *P* =0.020) and pN stage (HR [CI] 6.370 (1.384–29.323); *P* = 0.017). However, the surgical approach (robot or laparoscopy) was not associated with overall survival or disease-free survival or cumulative local recurrence (all *P* > 0.05; Supplementary Tables 5 & 6).

## Discussion

Left colectomy is a major method for curing left colonic cancer. Despite the controversy, three techniques including open, laparoscopic, and robotic surgery for left colectomy are commonly used in clinical practice. This retrospective analysis of three techniques for left colectomy demonstrated similar long-term oncologic and survival outcomes but greatly improved perioperative clinical outcomes and decreased occurrence of short-term complications for MIS including laparoscopic or robotic surgery. A large number of clinical trials have shown that short-term outcomes of MIS in CRC were better than that of open surgery [23, 24] but without compromising survival outcomes [9, 25]. Our result also confirmed that MIS, in comparison with OS, was associated with better intraoperative and perioperative clinical outcomes, without compromising the oncology survival outcomes.

The clinical value of these three surgical techniques for left colectomy is dependent on oncology survival outcomes which are significantly influenced by pathologic parameters such as the number of retrieved lymph nodes and surgical margin status [26]. The average number of retrieved lymph nodes in the RS group was no less than 12 lymph nodes which were recommended for examination to ensure complete resection and adequate staging [27] and was comparable to the OS and LS groups. No positive margins were found in the three groups, but an anastomotic recurrence was found in the LS group with a survival time of more than 36 months.

Long-term survival is the gold index to evaluate the clinical application in cancer. For left colectomy for radical treatment of CRC, our results showed that overall and disease-free survival rate had no significant difference among OS, LS, and RS, indicating its clinical applicability. Similar survival outcomes among various surgical methods had been also verified. A single-center study [28] showed similar long-term oncologic outcomes in patients undergoing robot-assisted surgery for sigmoid cancer, with no significant differences in the 3-year overall survival rate and 3-year disease-free survival rate. Pinar et al. [29] analyzed 5978 patients for colon cancer and 3206 for rectal cancer and revealed a comparable 3-year disease-free survival rate. However, Mirkin et al. [30] found that RS was associated with improved survival in stage II and III diseases compared with LS. Additionally, according to our analysis by TNM stage, no significant differences were observed between the RS group and the LS group in overall survival rate and, except for stage II, in disease-free survival rate before PSM. The difference of stage II before PSM may be associated with different rates of patients with perineural or lymphovascular invasion at stage II. Indeed, after eliminating the confounding factors by PSM, our study showed RS showed a similar survival compared with LS, indicating the feasibility of RS in colonic cancer.

RS is considered to reduce trauma and improve quality of life while ensuring radical resection [31]. Our study showed that RS showed shorter operation time and LOS for patients without complications, but both exhibited similar perioperative and long-term postoperative complications. Bhama et al. [32] and Ng et al. [14] verified that short-term clinical outcomes for RS were better than those for LS in CRC. However, a multicenter randomized clinical trial, the ROLARR study [33], revealed that robotic surgery did not gain a clinical advantage over conventional laparoscopic surgery in the short-term clinical outcome. The shorter operation time for RS may be as follows: compared with conventional laparoscopic technique, the robotic technique theoretically shows advantages in CRC surgery such as better visualization of fine anatomical structures, the ability to perform a finer and more dexterous dissection of splenic flexure [34], which may be the reason for the differential conversion rate between the RS group and the LS group. Additionally, unlike laparoscopic surgery, which requires tacit cooperation between the chief surgeon and his assistants, robotic surgery is mainly performed by the chief surgeon and the surgical scope is easily exhibited by robotic arms, which allows for a faster and smoother operation for experienced and skilled surgeons.

## Conclusions

Among the three techniques for radical left colectomy, MIS including laparoscopic and robotic surgery had significant advantages over open surgery in short-term outcomes, and no significant differences were found in overall, disease-free survival, local recurrence, and distant metastasis incidence rates. Moreover, RS shows better perioperative clinical outcomes but without compromising survival compared with LS.

However, this study is a retrospective analysis. The analysis of consecutive patients does represent the “real world” at our large-scale center. Some limitations were inherent to a retrospective analysis, including patients’ selection, inclusion, and recall bias. The findings also need to be further validated by a larger multicenter prospective randomized trial and longer follow-up survival data.

## Supplementary Information

**Additional file 1: Supplementary Figure 1.** Illustrations for the length of bowel resection, location of related arteries ligation and scope of the lymph node dissection for radical left colectomy for tumours located (A) at the distal 1/3 of the transverse colon (TC) and splenic flexure (SF), (B) at the upper segment of descending colon (UDC), (C) at the lower segment of descending colon (LDC).

**Additional file 2: Supplementary Figure 2.** (A1&B1&C1&D1): The values of hematology examination including of RBC (A1), WBC (B1), TP (C1) and ALB (D1) in OS, LS and RS groups were compared between pre-operation and post-operation by the Kruskal-Wallis test. (A2&B2&C2&D2): The changes of hematology examination including of RBC (A2), WBC (B2), TP (C2) and ALB (D2) from pre-operation to post-operation were compared among OS, LS and OS by the Kruskal-Wallis test. The Delta (Δ) is the postoperative value minus the preoperative value. RS (robotic surgery), LS (laparoscopic surgery), OS (open surgery).

**Additional file 3: Supplementary Figure 3.** Kaplan–Meier survival curves and Cumulative incidence curves (Before Propensity-score matched cohort). **(**A) Kaplan–Meier survival curves for overall survival rates according to TNM stage. (A1) All stages; (A2) Stage I; (A3) Stage II; (A4) Stage III. (B) Kaplan–Meier survival curves for disease-free survival rates according to TNM stage. (B1) All stages; (B2) Stage I; (B3) Stage II; (B4) Stage III. (C) Cumulative incidence curves of local recurrence rates. (D) Cumulative incidence curves of distant metastasis rates.

**Additional file 4: Supplementary Table 1**. Demographics and clinical characteristics of three groups. **Supplementary Table 2.** Demographics, clinical characteristics and pathologic outcomes before and after PSM. **Supplementary Table 3.** Perioperative clinical outcomes and short-term oncology outcomes (propensity-score matched cohort). **Supplementary Table 4.** Perioperative and long-term postoperative complications (propensity-score matched cohort). **Supplementary Table 5.** Prognostic factors of 3-year survival and local recurrence by univariate analysis (propensity-score matched cohort). **Supplementary Table 6.** Prognostic factors of 3-year survival and local recurrence by multivariate analysis (propensity-score matched cohort).

## Abbreviations

RS: Robotic surgery
LS: Laparoscopic surgery
OS: Open surgery
BMI: Body mass index
ASA: American Society of Anesthesiology
CRC: Colorectal cancer
CEA: Carcinoembryonic antigen
CA 199: Glucoprotein antigen 199
RBC: Red blood cells
WBC: White blood cells
TP: Total protein
ALB: Albumin
Hb: Hemoglobin
CCI: Charlson Comorbidity Index
SEMS: Self-expanding metal stent
LOS: Length of stay
TC: The distal 1/3 of the transverse colon
SF: Splenic flexure
UDC: Upper segment of descending colon
LDC: Lower segment of descending colon
pTNM: Pathological tumor-node-metastasis
