# Supplementary figures and images for: Comparison of open, laparoscopic, and robotic left colectomy for radical treatment of colon cancer: a retrospective analysis in a consecutive series of 211 patients
Source: World J Surg Oncol. 2022 Oct 18;20:345. doi: 10.1186/s12957-022-02796-8 (PMC9578184; doi:10.1186/s12957-022-02796-8)

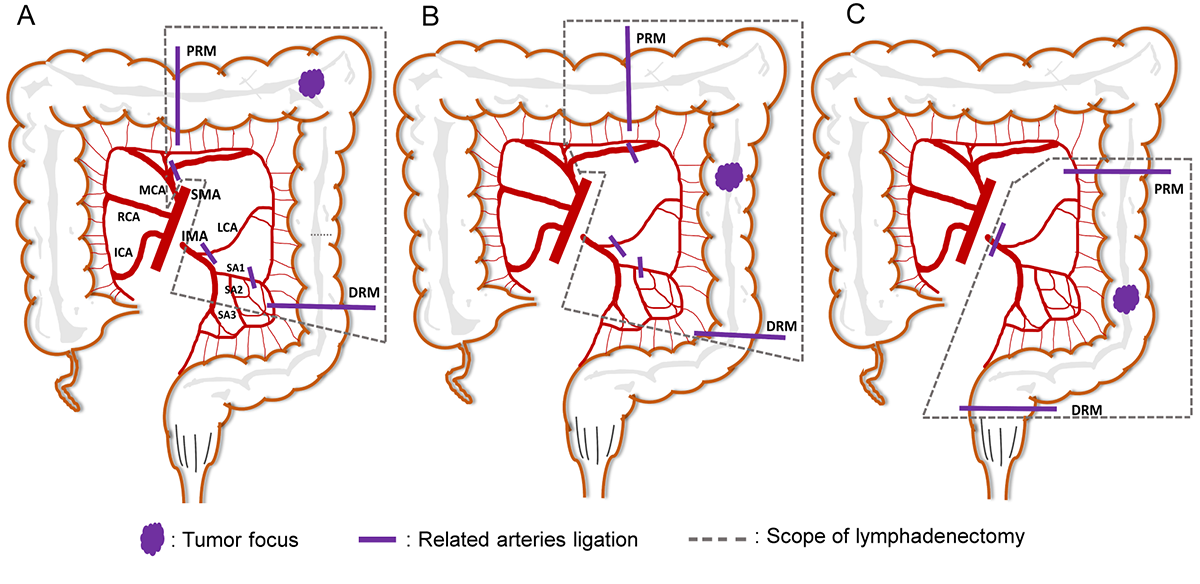

Supplement: Supplementary file 1 — Additional file 1: Supplementary Figure 1. Illustrations for the length of bowel resection, location of related arteries ligation and scope of the lymph node dissection for radical left colectomy for tumours located (A) at the distal 1/3 of the transverse colon (TC) and splenic flexure (SF), (B) at the upper segment of descending colon (UDC), (C) at the lower segment of descending colon (LDC). [file 12957_2022_2796_MOESM1_ESM.tif]

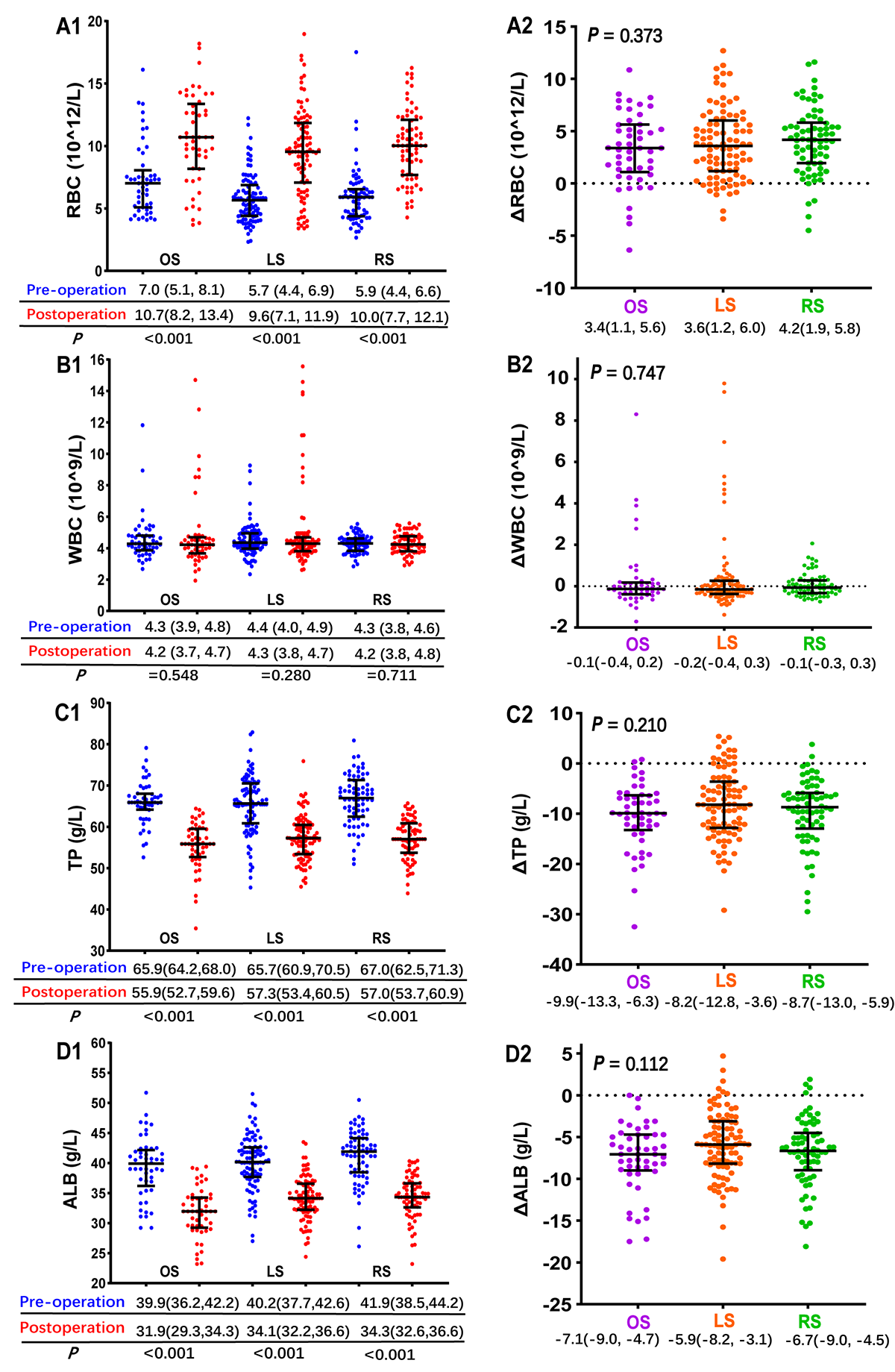

Supplement: Supplementary file 2 — Additional file 2: Supplementary Figure 2. (A1&B1&C1&D1): The values of hematology examination including of RBC (A1), WBC (B1), TP (C1) and ALB (D1) in OS, LS and RS groups were compared between pre-operation and post-operation by the Kruskal-Wallis test. (A2&B2&C2&D2): The changes of hematology examination including of RBC (A2), WBC (B2), TP (C2) and ALB (D2) from pre-operation to post-operation were compared among OS, LS and OS by the Kruskal-Wallis test. The Delta (Δ) is the postoperative value minus the preoperative value. RS (robotic surgery), LS (laparoscopic surgery), OS (open surgery). [file 12957_2022_2796_MOESM2_ESM.tif]

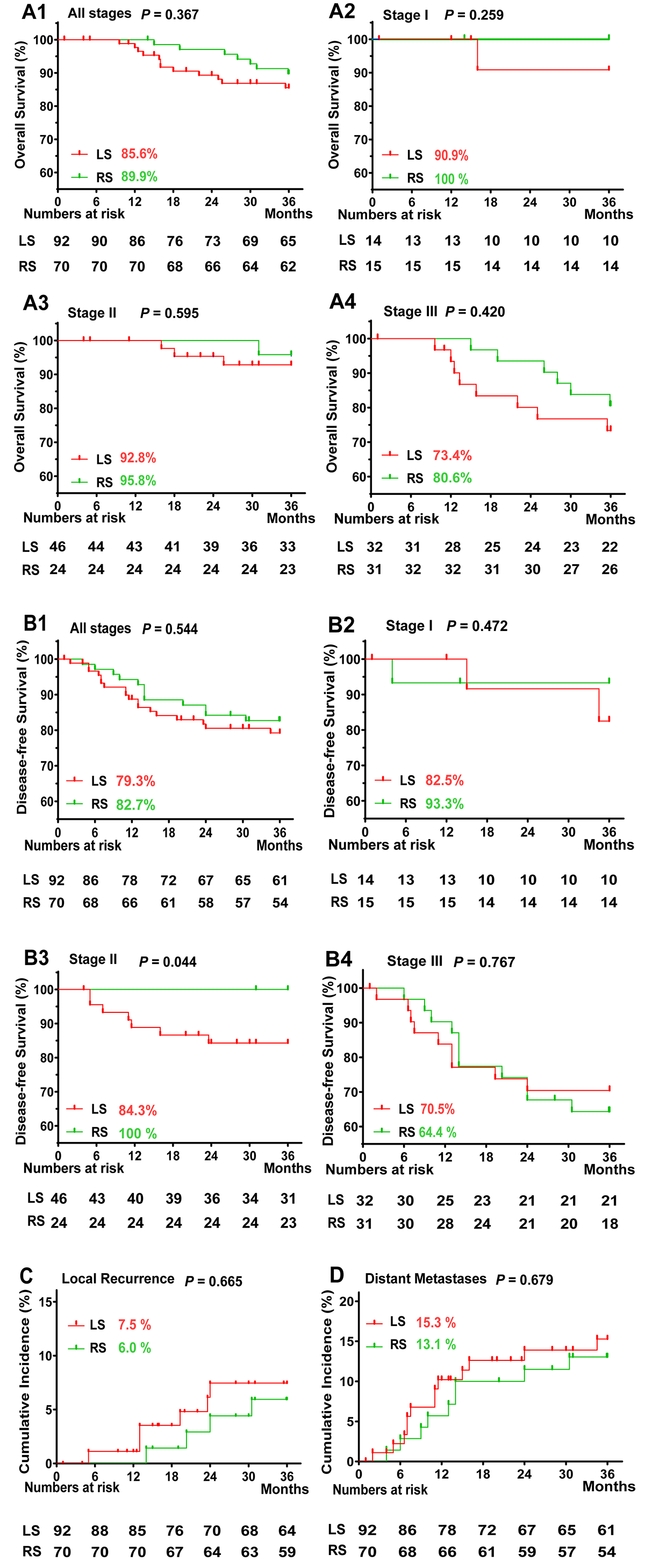

Supplement: Supplementary file 3 — Additional file 3: Supplementary Figure 3. Kaplan–Meier survival curves and Cumulative incidence curves (Before Propensity-score matched cohort). (A) Kaplan–Meier survival curves for overall survival rates according to TNM stage. (A1) All stages; (A2) Stage I; (A3) Stage II; (A4) Stage III. (B) Kaplan–Meier survival curves for disease-free survival rates according to TNM stage. (B1) All stages; (B2) Stage I; (B3) Stage II; (B4) Stage III. (C) Cumulative incidence curves of local recurrence rates. (D) Cumulative incidence curves of distant metastasis rates. [file 12957_2022_2796_MOESM3_ESM.tif]
