# Supplementary material for: Comparison of open, laparoscopic, and robotic left colectomy for radical treatment of colon cancer: a retrospective analysis in a consecutive series of 211 patients
Source: World J Surg Oncol. 2022 Oct 18;20:345. doi: 10.1186/s12957-022-02796-8 (PMC9578184; doi:10.1186/s12957-022-02796-8)
Supplement: Supplementary file 4 — Additional file 4: Supplementary Table 1. Demographics and clinical characteristics of three groups. Supplementary Table 2. Demographics, clinical characteristics and pathologic outcomes before and after PSM. Supplementary Table 3. Perioperative clinical outcomes and short-term oncology outcomes (propensity-score matched cohort). Supplementary Table 4. Perioperative and long-term postoperative complications (propensity-score matched cohort). Supplementary Table 5. Prognostic factors of 3-year survival and local recurrence by univariate analysis (propensity-score matched cohort). Supplementary Table 6. Prognostic factors of 3-year survival and local recurrence by multivariate analysis (propensity-score matched cohort). [file 12957_2022_2796_MOESM4_ESM.doc]

**Supplementary table 1****. Demographics and clinical characteristics of three groupsssss**

| **Variables** | OS  N=49 | LS  N=92 | RS  N=70 | *P* |
| --- | --- | --- | --- | --- |
| Age (years) | 57.0±12.3 | 58.9±13.7 | 60.4±12.7 | 0.363**^§^** |
| Sex (male) | 32 (65.3%) | 53 (57.6%) | 39 (55.7%) | 0.553**^†^** |
| BMI (kg/m^2^) | 22.7±3.4 | 22.4±2.8 | 22.6±3.1 | 0.934**^§^** |
| ASA score, n. (%) |  |  |  | 0.525**^‡^** |
| 1 | 0 (0.0%) | 1 (1.1%) | 0 (0.0%) |  |
| 2 | 20 (40.8%) | 41 (44.6%) | 24 (34.3%) |  |
| 3 | 29 (59.2%) | 50 (54.3%) | 46 (65.7%) |  |
| Previous abdominal surgery, n. (%) | 16 (32.7%) | 17 (18.5%) | 12 (17.1%) | 0.085**^†^** |
| Smoking and drinking history, n. (%) | 18 (36.7%) | 30 (32.6%) | 18 (25.7%) | 0.414**^†^** |
| Family history of CRC, n. (%) | 5 (10.2%) | 7 (7.6%) | 9 (12.9%) | 0.534**^‡^** |
| Preoperative hematology examination |  |  |  |  |
| CEA (ng/ml) | 6.6±8.9 | 6.7±12.4 | 5.0±10.9 | s0.068**^§^** |
| CA199 (ng/ml) | 24.4±47.6 | 18.1±24.5 | 16.4±28.5 | 0.316**^§^** |
| RBC (10^12/L) | 7.3±2.9 | 5.8±2.0 | 5.9±2.2 | **0.002^§^** |
| WBC (10^9/L) | 4.6±1.4 | 4.5±1.1 | 4.3±0.6 | 0.370**^§^** |
| Hb (g/dL) | 12.2±2.2 | 11.9±2.6 | 12.1±2.0 | 0.765**^§^** |
| TP (g/L) | 66.0±5.1 | 65.4±7.0 | 66.6±6.1 | 0.487**^§^** |
| ALB (g/L) | 39.1±4.9 | 40.0±4.6 | 41.2±4.3 | **0.022^§^** |
| Lymph node metastasis*****, no. (%) | 20 (40.8%) | 32 (34.8%) | 16 (22.9%) | 0.093**^†^** |
| CCI score, no. (%) |  |  |  | 0.720**^‡^** |
| 0 | 34 (69.4%) | 54 (58.7%) | 43 (61.4%) |  |
| 1-2 | 13 (26.5%) | 35 (38.0%) | 25 (35.7%) |  |
| 3-4 | 2 (4.1%) | 3 (3.3%) | 2 (2.9%) |  |
| With Comorbidities, n. (%) | 15 (30.6%) | 38 (41.3%) | 27 (38.6%) | 0.456**^†^** |
| History of SEMS insertion**^#^**, n. (%) | 6 (12.2%) | 7 (7.6%) | 4 (5.6%) | 0.422**^‡^** |
| Postoperative chemotherapy, n. (%) | 15 (30.6%) | 23 (25.0%) | 9 (12.9%) | 0.055**^†^** |

Values were expressed as mean (SD = standard deviation) or n (%).

**Abbreviation:** RS, robotic surgery; LS (laparoscopic surgery), OS (open surgery), BMI (body mass index), ASA (American Society of Anesthesiology), CRC (colorectal cancer), CEA (carcinoembryonic antigen), CA 199 (glucoprotein antigen 199), RBC (red blood cells), WBC (white blood cells), Hb (Hemoglobin), TP (total protein), ALB (albumin), CCI (Charlson Comorbidity Index), SEMS (self-expanding metal stent).

*****CT scan suggested regional lymph node metastasis before surgery.

**^#^**SEMS insertion was performed before left colectomy due to intestinal obstruction.

**^§^** Kruskal-Wallis test, **^†^** Pearson’s Chi squared test, **^‡^** Fisher’s exact test

**Supplementary Table 2. Demographics, clinical characteristics and pathologic outcomes before and after PSM**

| **Variables** | Before PSM | | *P* | After PSM | | *P* |
| --- | --- | --- | --- | --- | --- | --- |
|  | LS (N=92) | RS (N=70) |  | LS (N=52) | RS (N=52) |  |
| Age (years) | 58.9±13.7 | 60.4±12.7 | 0.463§ | 61.2±13.0 | 59.7±13.7 | 0.578§ |
| Sex (male) | 53 (57.6%) | 39 (55.7%) | 0.809† | 30 (57.7%) | 29 (55.8%) | 0.843† |
| BMI (kg/m^2^) | 22.4±2.8 | 22.6±3.1 | 0.695§ | 22.4±3.1 | 22.4±3.1 | 0.932§ |
| ASA score, n. (%) |  |  | 0.222‡ |  |  | 0.839† |
| 1 | 1 (1.1%) | 0 (0.0%) |  | 0 (0.0%) | 0 (0.0%) |  |
| 2 | 41 (44.6%) | 24 (34.3%) |  | 19 (36.5%) | 20 (38.5%) |  |
| 3 | 50 (54.3%) | 46 (65.7%) |  | 29 (63.5%) | 28 (61.5%) |  |
| Previous abdominal surgery, n. (%) | 17 (18.5%) | 12 (17.1%) | 0.826† | 11 (21.2%) | 9 (17.3%) | 0.804† |
| Smoking and drinking history, n. (%) | 30 (32.6%) | 18 (25.7%) | 0.420† | 13 (25.0%) | 15 (28.8%) | 0.658† |
| Family history of CRC, n. (%) | 7 (7.6%) | 9 (12.9%) | 0.267‡ | 4 (7.7%) | 5 (9.6%) | 1.000‡ |
| Preoperative hematology examination |  |  |  |  |  |  |
| CEA (ng/ml) | 6.7±12.4 | 5.0±10.9 | 0.373§ | 4.7±10.5 | 5.6±12.6 | 0.708§ |
| CA199 (ng/ml) | 18.1±24.5 | 16.4±28.5 | 0.682§ | 14.9±11.2 | 16.4±31.1 | 0.745§ |
| RBC (10^12/L) | 5.8±2.0 | 5.9±2.2 | 0.782§ | 5.6±2.0 | 5.9±2.3 | 0.407§ |
| WBC (10^9/L) | 4.5±1.1 | 4.3±0.6 | 0.049§ | 4.5±1.0 | 4.3±0.6 | 0.245§ |
| Hb (g/dL) | 11.9±2.6 | 12.1±2.0 | 0.619§ | 12.2±2.9 | 12.1±1.9 | 0.798§ |
| TP (g/L) | 65.4±7.0 | 66.6±6.1 | 0.261§ | 66.0±7.2 | 66.0±5.9 | 0.989§ |
| ALB (g/L) | 40.0±4.6 | 41.2±4.3 | 0.111§ | 40.4±4.8 | 40.8±4.5 | 0.688§ |
| Lymph node metastasis*****, no. (%) | 32 (34.8%) | 16 (22.9%) | 0.119† | 14 (26.9%) | 15 (28.8%) | 0.827† |
| CCI score, no. (%) |  |  | 0.938‡ |  |  | 0.880‡ |
| 0 | 54 (58.7%) | 43 (61.4%) |  | 28 (53.8%) | 29 (55.8%) |  |
| 1-2 | 35 (38.0%) | 25 (35.7%) |  | 23 (44.2%) | 21 (40.4%) |  |
| 3-4 | 3 (3.3%) | 2 (2.9%) |  | 1 (1.9%) | 2 (3.8%) |  |
| With Comorbidities, n. (%) | 38 (41.3%) | 27 (38.6%) | 0.749† | 24 (46.2%) | 23 (44.2%) | 0.844† |
| History of SEMS insertion**^#^**, n. (%) | 7 (7.6%) | 4 (5.6%) | 0.758‡ | 4 (7.7%) | 4 (7.7%) | 1.000‡ |
| Postoperative chemotherapy, n. (%) | 23 (25.0%) | 9 (12.9%) | 0.073† | 9 (17.3%) | 8 (15.4%) | 0.791† |
| Tumour location |  |  | 0.063† |  |  | 0.840† |
| TC&SF, n (%) | 26 (28.3%) | 16 (22.9%) |  | 8 (15.4%) | 9 (17.3%) |  |
| UDC, n (%) | 29 (31.5%) | 21 (30.0%) |  | 18 (34.6%) | 20 (38.5%) |  |
| LDC, n (%) | 37 (40.2%) | 33 (47.1%) |  | 26 (50.0%) | 23 (44.2%) |  |
| With adenomatous polyps, n (%) | 31 (33.7%) | 14 (20.0%) | 0.076† | 17 (32.7%) | 11 (21.2%) | 0.269† |
| Neoplasm diameter (cm) | 5.1±1.8 | 5.0±1.8 | 0.243§ | 4.8±1.3 | 5.0±1.7 | 0.440§ |
| Tumour differentiation |  |  | 0.988‡ |  |  | 1.000‡ |
| Well, n (%) | 10 (10.9%) | 8 (11.4%) |  | 6 (11.5%) | 6 (11.5%) |  |
| Moderate, n (%) | 69 (75.0%) | 54 (77.1%) |  | 38 (73.1%) | 39 (75.0%) |  |
| Poor, n (%) | 8 (8.7%) | 5 (7.1%) |  | 4 (7.7%) | 4 (7.7%) |  |
| Mucinous, n (%) | 5 (5.4%) | 3 (4.3%) |  | 4 (7.7%) | 3 (5.8%) |  |
| pTNM stage |  |  | 0.172† |  |  | 0.800† |
| I, n (%) | 14 (15.2%) | 15 (21.4%) |  | 8 (15.4%) | 10 (19.2%) |  |
| II, n (%) | 45 (48.9%) | 24 (34.3%) |  | 24 (46.2%) | 21 (40.4%) |  |
| III, n (%) | 33 (35.9%) | 31 (44.3%) |  | 20 (38.5%) | 21 (40.4%) |  |
| pT stage |  |  | 0. 455‡ |  |  | 0.867‡ |
| T1, n (%) | 10 (10.9%) | 10 (14.3%) |  | 7 (13.5%) | 7 (13.5%) |  |
| T2, n (%) | 4 (4.3%) | 5 (7.1%) |  | 1 (1.9%) | 3 (5.8%) |  |
| T3, n (%) | 11 (12.0%) | 4 (5.7%) |  | 4 (7.7%) | 3 (5.8%) |  |
| T4a, n (%) | 62 (72.8%) | 51 (72.9%) |  | 40 (76.9%) | 39 (75.0%) |  |
| pN stage |  |  | 0.282† |  |  | 0.887‡ |
| N0, n (%) | 59 (64.1%) | 39 (55.7%) |  | 32 (61.5%) | 31 (59.6%) |  |
| N1, n (%) | 21 (22.8%) | 24 (34.3%) |  | 14 (26.9%) | 16 (30.8%) |  |
| N2, n (%) | 12 (13.0%) | 7 (10.0%) |  | 6 (11.5%) | 5 (9.6%) |  |
| With lymph node metastasis, n (%) | 32 (34.8%) | 31 (44.3%) | 0.256† | 19 (36.5%) | 21 (40.4%) | 0.687† |
| With lymphovascular invasion, n (%) | 14 (15.2%) | 10 (14.3%) | 0.869† | 6 (11.5%) | 8 (15.4%) | 0.566† |
| With extranodal tumor deposits, n (%) | 14 (15.2%) | 13 (18.6%) | 0.671† | 9 (17.3%) | 11 (21.2%) | 0.619† |
| With perineural invasion, n (%) | 34 (37.0%) | 24 (34.3%) | 0.744† | 21 (40.4%) | 18 (34.6%) | 0.686† |

Values were expressed as mean (SD = standard deviation) or n (%).

**Abbreviation:** BMI (body mass index), ASA (American Society of Anesthesiology), CRC (colorectal cancer), CEA (carcinoembryonic antigen), CA 199 (glucoprotein antigen 199), RBC (red blood cells), WBC (white blood cells), Hb (Hemoglobin),TP (total protein), ALB (albumin), CCI (Charlson Comorbidity Index), SEMS (self-expanding metal stent), TC (the distal 1/3 of the transverse colon), SF (splenic flexure), UDC (upper segment of descending colon), LDC (lower segment of descending colon), pTNM (pathological tumor-node-metastasis)

*****CT scan suggested regional lymph node metastasis before surgery.

**^#^**SEMS insertion was performed before left colectomy due to intestinal obstruction.

**^§^** T test, **^†^** Pearson’s Chi squared test, **^‡^** Fisher’s exact test.

Supplementary Table 3. Perioperative clinical outcomes and short-term oncology outcomes (propensity-score matched cohort)

| **Variables** | Laparoscopic  N=52 | Robotic  N=52 | *P* |
| --- | --- | --- | --- |
| Operation time (min) | 188.3±46.0 | 149.2±41.7 | **<0.001**^§^ |
| Blood loss (ml) | 150.9±66.9 | 133.5±70.1 | 0.198**^§^** |
| Time to first bowel movement (h) | 38.8±15.5 | 32.8±15.8 | 0.053**^§^** |
| Time to first flatus (h) | 64.2±16.3 | 62.3±7.1 | 0.456§ |
| Time to first liquid diet (h) | 86.2±21.8 | 80.7±12.2 | 0.153§ |
| LOS for all patients (d) | 8.7±4.7 | 7.8±3.4 | 0.288§ |
| LOS for patients without complications (d) | 7.2±0.7 | 6.8±0.8 | **0.005^§^** |
| LOS for patients with complications (d) | 14.7±8.6 | 15.8±5.3 | 0.776**^§^** |
| Number of lymph node | 13.4±6.4 | 14.0±4.9 | 0.583**^§^** |
| Positive resection margin, n (%) | 0 (0.0%) | 0 (0.0%) | / |

Values were expressed as mean (SD = standard deviation) or n (%)

Abbreviation: LOS (length of stay)

**^§^** T test, **^†^** Pearson’s Chi squared test, **^‡^** Fisher’s exact test

**Supplementary Table 4. Perioperative and long-term postoperative complications (propensity-score matched cohort)**

| **Variables** | Laparoscopic  N=52 | Robotic N=52 | *P* |
| --- | --- | --- | --- |
| Perioperative complications***** |  |  |  |
| Mortality, n (%) | 0 (0.0%) | 0 (0.0%) | / |
| Reoperation, n. (%) | 0 (0.0%) | 0 (0.0%) | / |
| Readmission, n. (%) | 0 (0.0%) | 0 (0.0%) | / |
| Overall morbidity, n (%) | 10 (19.2%) | 6 (11.5%) | 0.277**^‡^** |
| Grade I/II complications, n (%) | 10 (18.2%) | 6 (15.9%) | 0.277**^‡^** |
| Wound infection | 5 (9.6%) | 4 (7.7%) | 1.000**^‡^** |
| Intra-abdominal infection | 1 (1.9%) | 0 (0.0%) | 1.000**^‡^** |
| Ileus | 1 (1.9%) | 1 (1.9%) | 1.000**^‡^** |
| Acute pneumonia | 0 (0.0%) | 1 (1.9%) | 1.000**^‡^** |
| Fever of unknown origin | 1 (1.9%) | 0 (0.0%) | 1.000**^‡^** |
| Anastomotic hemorrhage | 1 (1.9%) | 0 (0.0%) | 1.000**^‡^** |
| Anastomotic leak | 1 (1.9%) | 0 (0.0%) | 1.000**^‡^** |
| Blood transfusion due to anemia | 0 (0.0%) | 0 (0.0%) | / |
| Grade III/IV complications, n (%) | 0 (0.0%) | 0 (0.0%) | / |
| Wound dehiscence (fascia) | 0 (0.0%) | 0 (0.0%) | / |
| Intra-abdominal infection and effusion | 0 (0.0%) | 0 (0.0%) | / |
| Ileus | 0 (0.0%) | 0 (0.0%) | / |
| Acute liver failure | 0 (0.0%) | 0 (0.0%) | / |
| Grade V complications/Mortality, n (%) | 0 (0.0%) | 0 (0.0%) | / |
| Major perioperative complications, n (%) | 0 (0.0%) | 0 (0.0%) | / |
| Long-term postoperative complications**^#^** |  |  |  |
| Overall morbidity, n (%) | 1 (1.9%) | 2 (3.8%) | 1.000**^‡^** |
| Grade I/II complications, n (%) | 0 (0.0%) | 1 (1.9%) | 1.000**^‡^** |
| Incisional hernia | 0 (0.0%) | 0 (0.0%) | / |
| Ileus | 0 (0.0%) | 1 (1.9%) | 1.000**^‡^** |
| Grade III/IV complications, n (%) | 1 (1.9%) | 1 (1.9%) | 1.000**^‡^** |
| Incisional hernia | 0 (0.0%) | 0 (0.0%) | / |
| Adhesion | 1 (1.9%) | 0 (0.0%) | 1.000**^‡^** |
| Anastomotic stricture | 0 (0.0%) | 1 (1.9%) | 1.000**^‡^** |
| Grade V complications/Mortality, n (%) | 0 (0.0%) | 0 (0.0%) | / |
| Major long-term complications, n (%) | 1 (1.9%) | 1 (1.9%) | 1.000**^‡^** |

Values were expressed as n (%).

Major complications were defined as complications with a grade III and higher of the Clavien–Dindo classification.

*****Complications within 30 days from operation date

**^#^**New complications 30 days after operation date

**^§^** T test, **^†^** Pearson’s Chi squared test, **^‡^** Fisher’s exact test

Supplementary Table 5. Prognostic factors of 3-year survival and local recurrence by univariate analysis (propensity-score matched cohort)

| **Variables** | N=104 | Overall Survival (%) | *P* | Disease-free  Survival (%) | *P* | Cumulative  local recurrence (%) | *P* |
| --- | --- | --- | --- | --- | --- | --- | --- |
| Age (years) |  |  | 0.221 |  | 0.478 |  | 0.659 |
| ≤65 | 60 | 91.4 |  | 84.7 |  | 6.4 |  |
| > 65 | 44 | 82.9 |  | 77.8 |  | 7.6 |  |
| Sex |  |  | 0.295 |  | 0.918 |  | 0.624 |
| Male | 59 | 91.0 |  | 81.9 |  | 7.4 |  |
| Female | 45 | 83.7 |  | 81.8 |  | 4.7 |  |
| BMI (kg/m^2^) |  |  | 0.912 |  | 0.411 |  | 0.253 |
| ≤25 | 84 | 87.5 |  | 80.2 |  | 7.6 |  |
| >25 | 20 | 89.5 |  | 89.5 |  | 0.0 |  |
| ASA score |  |  | 0.817 |  | 0.807 |  | 0.486 |
| 1 | 0 | / |  | / |  | / |  |
| 2 | 39 | 89.2 |  | 81.2 |  | 8.6 |  |
| 3 | 65 | 87.1 |  | 82.3 |  | 4.9 |  |
| CEA (ng/ml) |  |  | 0.054 |  | **0.009** |  | **0.010** |
| ≤ 6.5 | 89 | 90.4 |  | 85.7 |  | 3.7 |  |
| > 6.5 | 15 | 73.3 |  | 60.0 |  | 21.4 |  |
| CA199 (ng/ml) |  |  | 0.075 |  | 0.057 |  | 0.050 |
| ≤27 | 92 | 89.9 |  | 84.3 |  | 4.7 |  |
| > 27 | 12 | 70.0 |  | 60.0 |  | 20.0 |  |
| Surgical approach |  |  | 0.364 |  | 0.782 |  | 0.535 |
| Laparoscopy | 52 | 85.1 |  | 83.2 |  | 4.5 |  |
| Robot | 52 | 90.4 |  | 80.7 |  | 7.8 |  |
| Previous abdominal surgery |  |  | 0.089 |  | 0.133 |  | 0.893 |
| Yes | 20 | 100.0 |  | 94.4 |  | 5.6 |  |
| No | 84 | 85.1 |  | 79.1 |  | 6.4 |  |
| Smoking and drinking history |  |  | 0.427 |  | 0.182 |  | 0.201 |
| Yes | 28 | 92.1 |  | 73.2 |  | 11.5 |  |
| No | 76 | 86.2 |  | 85.0 |  | 4.3 |  |
| Family history of CRC |  |  | 0.287 |  | 0.720 |  | 0.452 |
| Yes | 9 | 100.0 |  | 88.9 |  | 0.0 |  |
| No | 95 | 86.7 |  | 81.4 |  | 6.9 |  |
| With Comorbidities |  |  | 0.111 |  | 0.256 |  | 0.587 |
| Yes | 47 | 81.7 |  | 77.3 |  | 4.9 |  |
| No | 57 | 92.7 |  | 85.5 |  | 7.5 |  |
| Postoperative adjuvant chemotherapy |  |  | 0.317 |  | 0.391 |  | 0.880 |
| Yes | 17 | 81.6 |  | 75.3 |  | 6.7 |  |
| No | 87 | 89.0 |  | 83.1 |  | 6.2 |  |
| Perioperative morbidity |  |  | 0.851 |  | 0.613 |  | 0.910 |
| Yes | 16 | 86.7 |  | 86.7 |  | 7.1 |  |
| No | 88 | 88.0 |  | 81.0 |  | 6.2 |  |
| Tumours location |  |  | 0.284 |  | 0.401 |  | 0.168 |
| TC&SF | 17 | 100.0 |  | 93.8 |  | 0.0 |  |
| UDC | 38 | 86.2 |  | 81.0 |  | 2.8 |  |
| LDC | 49 | 84.9 |  | 78.4 |  | 11.4 |  |
| With adenomatous polyps |  |  | 0.132 |  | 0.574 |  | 0.131 |
| Yes | 28 | 96.3 |  | 85.0 |  | 0.0 |  |
| No | 76 | 84.7 |  | 80.8 |  | 8.6 |  |
| Neoplasm diameter (cm) |  |  | 0.334 |  | 0.697 |  | 0.242 |
| ≤5 | 75 | 90.0 |  | 82.9 |  | 4.5 |  |
| >5 | 29 | 82.8 |  | 79.3 |  | 10.7 |  |
| Tumour differentiation |  |  | **0.017** |  | **0.004** |  | **0.011** |
| Well | 12 | 100.0 |  | 90.9 |  | 0.0 |  |
| Moderate | 77 | 88.9 |  | 86.3 |  | 4.2 |  |
| Poor | 8 | 87.5 |  | 62.5 |  | 14.3 |  |
| Mucinous | 7 | 57.1 |  | 42.9 |  | 33.3 |  |
| pTNM stage |  |  | 0.128 |  | **0.003** |  | 0.063 |
| I | 18 | 94.1 |  | 82.4 |  | 0.0 |  |
| II | 45 | 92.9 |  | 95.4 |  | 2.4 |  |
| III | 41 | 79.5 |  | 66.7 |  | 13.6 |  |
| pT stage |  |  | 0.287 |  | 0.085 |  | 0.570 |
| T1 | 14 | 100.0 |  | 92.3 |  | 0.0 |  |
| T2 | 4 | 75.0 |  | 50.0 |  | 0.0 |  |
| T3 | 7 | 100.0 |  | 100.0 |  | 0.0 |  |
| T4a | 79 | 85.2 |  | 80.2 |  | 8.3 |  |
| pN stage |  |  | **<0.001** |  | **0.001** |  | **<0.001** |
| N0 | 63 | 93.3 |  | 91.6 |  | 1.7 |  |
| N1 | 30 | 89.7 |  | 72.2 |  | 7.2 |  |
| N2 | 11 | 50.5 |  | 50.5 |  | 35.2 |  |
| Number of lymph node detected |  |  | 0.929 |  | 0.491 |  | 0.413 |
| ≤15 | 69 | 87.6 |  | 80.0 |  | 4.8 |  |
| >15 | 35 | 88.0 |  | 85.3 |  | 9.2 |  |
| With lymphovascular invasion |  |  | 0.617 |  | 0.491 |  | 0.366 |
| Yes | 14 | 83.3 |  | 75.5 |  | 0.0 |  |
| No | 90 | 88.4 |  | 82.7 |  | 7.1 |  |
| With extranodal tumor deposits |  |  | **0.046** |  | **<0.001** |  | **0.044** |
| Yes | 20 | 75.0 |  | 55.0 |  | 16.8 |  |
| No | 84 | 91.1 |  | 88.7 |  | 3.8 |  |
| With perineural invasion |  |  | 0.666 |  | 0.080 |  | 0.107 |
| Yes | 39 | 86.5 |  | 73.0 |  | 11.8 |  |
| No | 65 | 88.6 |  | 87.1 |  | 3.2 |  |

Abbreviation: BMI (body mass index), ASA (American Society of Anesthesiology), CRC (colorectal cancer), TC (the distal 1/3 of the transverse colon), SF (splenic flexure), UDC (upper segment of descending colon), LDC (lower segment of descending colon), pTNM (pathological tumor-node-metastasis)

*****The 3-year overall and disease-free survival rates and cumulative local recurrence were calculated by using the Kaplan–Meier method.

**Supplementary Table 6. Prognostic factors of 3-year survival and local recurrence by multivariate analysis (propensity-score matched cohort)**

| **Variables** | Overall Survival  HR (95% CI) | *P****** | Disease-free  Survival HR  (95% CI) | *P****** | Cumulative  local recurrence HR (95% CI) | *P****** |
| --- | --- | --- | --- | --- | --- | --- |
| CEA (ng/ml) | / | / | / | 0.719 | / | 0.923 |
| Tumour differentiation | 2.007  (1.009-3.993) | **0.047** | 2.665  (1.392-5.101) | **0.003** | 3.248  (1.203-8.772) | **0.020** |
| pTNM stage | / | / | / | 0.059 | / | / |
| pN stage | 2.920 (1.136-7.509) | **0.026** | 3.211 (1.154-8.934) | **0.025** | 6.370 (1.384-29.323) | **0.017** |
| With extranodal tumor deposits | / | 0.926 | 3.881  (1.046-14.403) | **0.043** | / | 0.775 |

*****Cox proportional hazards regression model.

CI indicates confidence interval; HR, hazard ratio.
